# Inferring tumor immune microenvironment -related risk states from pretreatment H&E pathomics and clinical biomarkers to predict checkpoint inhibitor pneumonitis in advanced NSCLC: a multicenter multimodal study

**DOI:** 10.3389/fimmu.2026.1792179

**Published:** 2026-02-19

**Authors:** Lei Yuan, Qi Wang, Fei Sun, Wenlong Yang, Jie Lei, Juan Liu, Yiwei Fan, Yibo Shan, Yi Lu, Yaojing Zhang, Yilun Wang, Jianwei Zhu, Lintao Guo, Wenxuan Chen, Shichun Lu, Hongcan Shi

**Affiliations:** 1 Department of Thoracic Surgery, Northern Jiangsu People’s Hospital Affiliated to Yangzhou University, Yangzhou, China; 2 Institute of Translational Medicine, Medical College, Yangzhou University, Yangzhou, China; 3 Department of Thoracic Surgery, The Affiliated Taizhou People’s Hospital of Nanjing Medical University, Taizhou, China; 4 Department of Pathology, Northern Jiangsu People’s Hospital Affiliated to Yangzhou University, Yangzhou, China; 5 Department of Radiology, The Fourth People’s Hospital of Lianyungang, Affiliated with Kangda College of Nanjing Medical University, Lianyungang, China

**Keywords:** checkpoint inhibitor pneumonitis, computational pathology, multi-instance learning, NSCLC, pathomics, tumor immune microenvironment

## Abstract

**Background:**

Checkpoint inhibitor pneumonitis (CIP) is a rare but potentially fatal immune-related adverse event (irAE) that can interrupt immune checkpoint blockade in non-small cell lung cancer (NSCLC). With no validated pretreatment biomarkers and a diagnosis largely made by exclusion, upfront risk stratification is required. Recent advances in artificial intelligence (AI)-driven pathomics have made it feasible to infer tumor immune microenvironment (TIME)-relevant risk states in patients with NSCLC. Accordingly, we leveraged hematoxylin and eosin(H&E)-based digital pathomics combined with clinical variables to interrogate the TIME in patients who developed CIP and to enable pretreatment and early prediction of CIP.

**Methods:**

In this retrospective study, 346 eligible patients from three hospitals were screened consecutively between January 2022 and January 2025. Patients were divided into CIP and non-CIP groups according to whether CIP occurred at a prespecified observation endpoint. We first developed a pathomics model that employed convolutional neural networks (CNNs) combined with multi-instance learning (MIL) to generate predictions at both the patch and whole slide image (WSI) levels on H&E-stained slides. Separately, we constructed a clinical model using logistic regression (LR) to process the structured clinical data accompanying each case. Subsequently, pathological and clinical information were integrated, where modeling was advanced from modality-specific feature learning to cross-modal representation learning, and final predictive modeling was completed. The predictive performance of different models was evaluated using the area under the Receiver Operating Characteristic (ROC) curve and benchmarked against unimodal models and standard ensemble methods.

**Results:**

When the models were evaluated across both internal validation and external test datasets, the pathomics model demonstrated noticeably stronger performance than the clinical approach, achieving area under the curve (AUC) scores of 0.916, 0.875(test 1), and 0.843(test 2), respectively, while the clinical model posted more modest results of 0.880, 0.569(test 1), and 0.594(test 2). The most significant outcome, however, emerged from the multimodal fusion model, which produced the strongest results of all, with performance metrics of 0.930, 0.919(test 1), and 0.905(test 2) in the validation and test phases, respectively.

**Conclusion:**

Pretreatment H&E-derived pathomics, integrated with baseline clinical biomarkers, enable accurate prediction of CIP risk in locally advanced or metastatic NSCLC. This framework supports proactive surveillance and individualized immune checkpoint inhibitor (ICI) strategies and provides a scalable route to decode TIME-relevant states from routine pathology.

## Introduction

Checkpoint inhibitor pneumonitis (CIP) has been reported to occur at an incidence of approximately 2.6%–33% (1, 2). However, it is not the most frequent immune-related adverse event (irAE). Nevertheless, it may represent one of the most severe irAEs observed in clinical practice. Grade 3–4 CIP, if not effectively treated, often disrupts ongoing antitumor immunotherapy in patients with advanced NSCLC (3). Early-stage or mild CIP may present without clinical manifestations. In contrast, severe CIP can lead to rapidly progressive dyspnea and respiratory failure (4). Currently, no CIP-specific serological markers have been established (2, 5). Thus, a practical strategy is urgently needed to stratify CIP risk before ICI initiation using available baseline clinical and pathological data from patients with locally advanced or metastatic NSCLC. High-risk patients can then be proactively managed. For example, Interleukin-6 (IL-6) receptor inhibitors (6) may be combined early to mitigate pulmonary toxicity driven by excessive cytokine release while preserving the antitumor efficacy of ICIs. In this way, individualized immunotherapy regimens could be optimized (7).

H&E-stained lung tumor slides were converted into whole-slide images (WSIs) (8). These WSIs contain valuable information regarding the tumor microenvironment (9). Consequently, Artificial intelligence (AI)-driven pathomics (10) analysis has shown potential across multiple tasks, including the quantification of the tumor immune microenvironment (TIME) (11), comprehensive image-based omics profiling, identification of prognostically relevant morphological features, and linkage of morphology with treatment response (12).

In this research endeavor, we developed and validated a multimodal framework that integrates pathological WSIs with baseline clinical data to predict the likelihood of CIP after ICIs therapy in locally advanced or metastatic NSCLC. Single-modality models were used as comparators. Through this design, multiscale insights into tumor heterogeneity and post-treatment adverse events were provided.

## Materials and methods

### Study design and setting

The data were collected from three medical centers, and approval was obtained from each institutional review board (2021ky211-1, KY 2024-093-01, and 2025LSYYXLL-P15). The participating centers included Northern Jiangsu People’s Hospital Affiliated to Yangzhou University (Center A), The Affiliated Taizhou People’s Hospital of Nanjing Medical University (Center B), and The Fourth People’s Hospital of Lianyungang (Center C). Patients with NSCLC who were administered Programmed death 1/ligand-1 (PD-1/L1) immune checkpoint inhibitors from January 2022 to January 2025 were included in this retrospective analysis. Cytotoxic T-lymphocyte-associated protein 4 (CTLA-4) inhibitors were not incorporated because no domestic approval was available at the time the project was initiated. Consequently, our analysis focused exclusively on PD-1/PD-L1 inhibitors. Eligible patients were treated with PD-1/PD-L1 inhibitors, including Pembrolizumab, Nivolumab, Tislelizumab, Sintilimab, and Durvalumab. Combination therapy included platinum-based chemotherapy plus pemetrexed (adenocarcinoma) or platinum-based chemotherapy plus paclitaxel (squamous NSCLC). The treatment plan followed the standard clinical protocols. Clinical data were extracted from the hospital information system (HIS), including age, gender, body mass index (BMI), smoking status, Eastern Cooperative Oncology Group (ECOG) performance status, lung cancer stage, pathological subtype, treatment regimen, driver oncogene alterations, PD-L1 expression, and laboratory test results. The full names of the selected laboratory variables are provided in the List of Glossary. For clinical variables with incomplete availability across centers (PD-L1 TPS), missing or “not detected” entries were encoded as an explicit standalone category rather than being imputed so that the model could handle real-world testing patterns without introducing imputation bias.

### Definition and adjudication of CIP

At the beginning of the study, the follow-up time was determined to be 12 months after the first medication of ICI, and this time point was used as the end point of observation. During the observation period, continuous evaluation was performed to confirm whether the patient had CIP, and the patients were divided into CIP and non-CIP groups. The diagnosis of CIP was established according to the current guidelines and consensus statements. In practice, the diagnostic framework we applied included five main criteria: (1) a history of ICI treatment in patients with advanced tumors; (2) new-onset cough or dyspnea after ICI therapy or worsening of pre-existing respiratory symptoms; (3) chest CT scans revealing new inflammatory changes, such as ground-glass opacities, consolidation, or interstitial pneumonitis patterns; (4) cases should demonstrate poor response to antibiotic therapy but noticeable improvement with corticosteroids; and (5) CIP was considered a diagnosis of exclusion, where ICI exposure history, bronchoalveolar lavage (BAL) fluid differential cell counts, and microbiological testing to help exclude infection.

Possible differential diagnoses that need to be considered include pulmonary infection, tumor progression, and other conditions, such as heart failure or pulmonary embolism, since these can all present with progressive dyspnea. The CIP assessments were performed by a multidisciplinary team of medical specialists that included pulmonologists, pathologists, radiation oncologists, and thoracic surgeons working together. They reviewed each case individually in detail until they reached a full consensus. Throughout the entire process, every step was conducted transparently and strictly followed the ethical guidelines established in the Declaration of Helsinki (as revised).

### Inclusion criteria

Individuals qualified for participation in the study if they met all the following prerequisites:

1. An ECOG performance status ranging from 0 to 3;
2. Measurable lung lesions found by routine chest high-resolution computed tomography (HRCT) examination are in line with the Response Evaluation Criteria in Solid Tumors (RECIST) v1.1 standard;
3. NSCLC confirmed by histopathology via biopsy or bronchoscopy, with clinical imaging and pathological staging consistent with the Tumor–Node–Metastasis (TNM), 8th edition stage IIIB–IV (13);
4. Having full access to comprehensive laboratory findings and imaging data is essential for assessment, which means that complete blood count (CBC), comprehensive metabolic panel (CMP), and H&E-stained tumor biopsy specimens were required before ICI initiation; follow-up HRCT was performed every 4–6 weeks after treatment initiation;
5. Complete follow-up records were obtained.

### Exclusion criteria

Patients were excluded if any of the following applied:

1. Suboptimal imaging quality that could affect assessment (motion or metal artifacts);
2. History of thoracic surgery;
3. Loss to follow-up after immunotherapy;
4. Incomplete acquisition of the required materials for pathological assessment, including H&E slides, laboratory findings, or imaging datasets.

**Figure 1:**
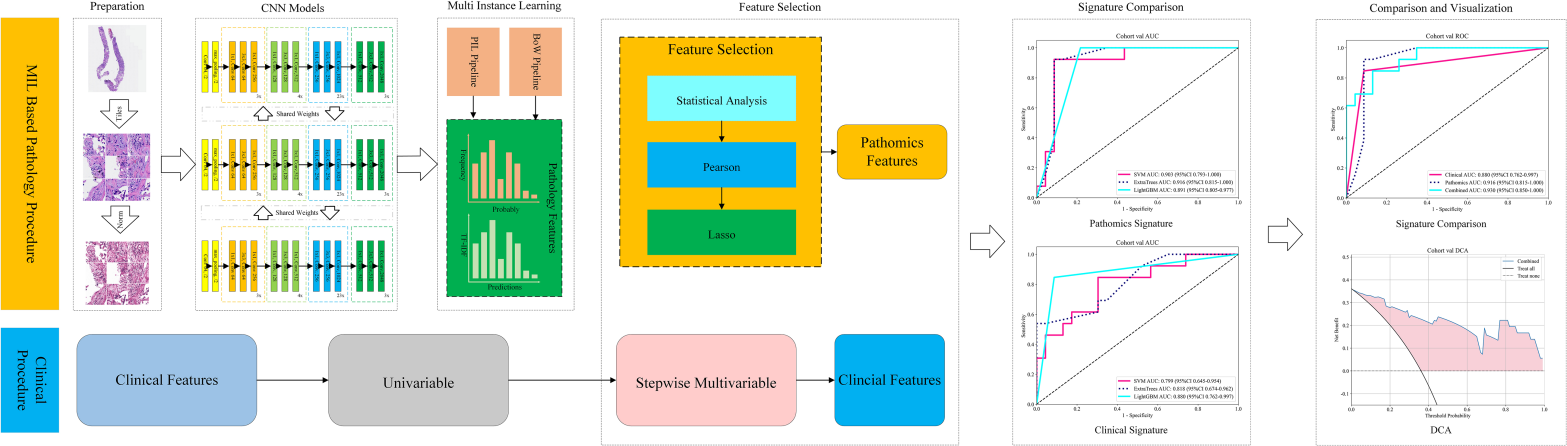
Workflow of this study.

The whole-slide images were partitioned into fixed-size, non-overlapping tiles to enable efficient computational processing, after which each tile was subjected to supervised patch-level classification using a convolutional neural network (CNN) (14). The network was trained to learn discriminative morphological signatures that distinguished the two diagnostic categories based on patch-level annotations. The resulting patch embeddings were subsequently integrated through a multi-instance learning (MIL) framework, which transformed the ensemble of patch-level outputs into slide-level descriptors suitable for downstream prediction tasks. Classical machine learning models were then trained on these aggregated descriptors to achieve case-level classification (15, 16). This streamlined workflow, shown in **Figure 1**, provides a scalable pipeline for interpreting patient-level predictions from raw WSI data.

In this retrospective study, 346 eligible patients from three hospitals were included (101 with CIP and 245 without CIP). The dataset was split according to the workflow shown in **Supplementary Figure 1**. After careful screening, we divided Dataset A into two parts: one was the training set, which contained 70% of the data; the other was the internal validation set, with the remaining 30% of the data. We used the method of stratified sampling so that the proportion of each kind of data could be kept the same. When developing the model, we used the method of “five-fold cross-validation” and combined with “grid search” to adjust the model step by step and find the best setting. After finding the best setting, we retrained the model with the entire training set and then tested it on additional external datasets (test 1 and test 2) to determine how the model behaves when it encounters unknown data. The external test cohorts were not accessed in any form during model development, including hyperparameter tuning, feature selection, normalization, threshold determination, or calibration.

### Multi instance learning based pathology procedure

#### Data processing

Multiple centers collected HE-stained sections of the scanned tumor puncture tissues of the enrolled patients. After the Motic Easyscan6 digital section scanning and application system, the Whole-slide images (WSIs) were obtained after 20X scanning with the preset parameters of the same machine. The Whole-slide images were then first partitioned into non-overlapping patches of 512 × 512 pixels at 20X magnification to accommodate their extremely large spatial dimensions. Patches consisting predominantly of black or white backgrounds were excluded to remove tiles with insufficient tissue content. This preprocessing workflow resulted in more than 12 million high-quality, tissue-containing patches. All procedures were executed on the OnekeyAI platform, utilizing OKT-crop_WSI2patch for patch extraction, OKT-patch2predict to filter background regions, and OKT-patch_normalize for stain normalization in order to mitigate inter-slide color variability.

#### Slice-level model training

**Figure 2:**
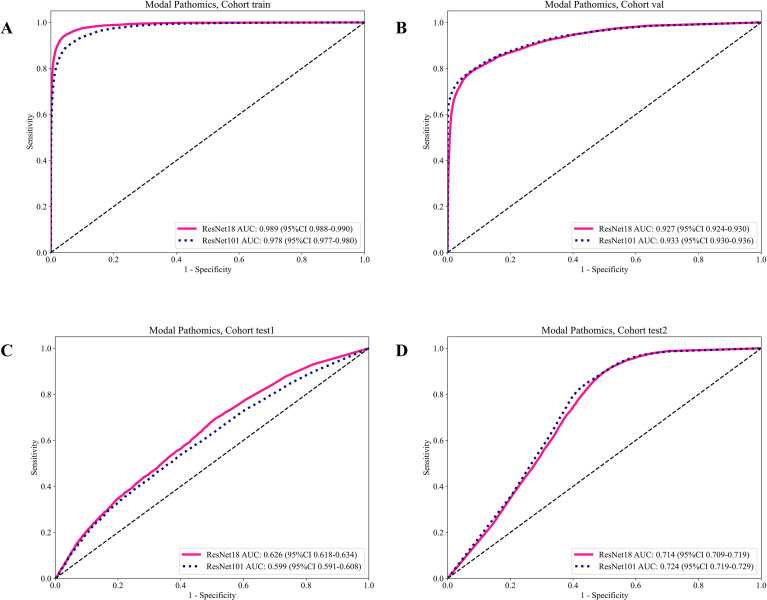
Showcases the ROC curves for each model’s performance on the different datasets: **(A)** training set; **(B)** validation set; **(C)** test 1 set; **(D)** test 2 set.

Each extracted slice was treated as an independent instance while maintaining the patient-level label to enable multi-instance learning. To evaluate backbone performance at the slice level, two representative CNN architectures—ResNet18 and ResNet101 (17)—were trained under an identical shared-parameter framework to enhance feature diversity and model robustness. Transfer learning was applied by initializing all models with ImageNet-pretrained weights, facilitating faster convergence and leveraging generalized hierarchical visual representations (**Figure 2**).

##### Data augmentation

To mitigate overfitting and improve generalizability, Z-score normalization was conducted across the RGB channels for each slice, and real-time data augmentation—including random cropping and horizontal/vertical flipping—was applied during training to enrich sample diversity. When it came to the validation and test sets, however, we stuck solely with normalization to maintain uniformity across all evaluations.

##### Data normalization and resizing

We adjust all pixel values to the range of [-1, 1], using the method of min-max normalization. In order to make them fit in with the CNN framework we chose, we use nearest-neighbor interpolation to resize the cut ROI pieces and unify them into a format of 224 × 224 pixels.

##### Training parameters

A cosine annealing learning-rate schedule was employed to dynamically adjust the learning rate throughout optimization:


$$\eta_{t}=\eta_{\min}^{i}+1/2(\eta_{\max}^{i}-\eta_{\min}^{i})(1+cos(T_{cur}/T_{i}\pi )),$$

where $\eta_{\min}^{i}=0$, $\eta_{\max}^{i}=0.01$, and $T_{i}=8$ denote the minimum and maximum learning rates and the total number of epochs, respectively. Model optimization was performed using stochastic gradient descent (SGD) with a softmax cross-entropy loss function.

#### Multi-instance learning for patient-level fusion

Following slice-level training, each slice generated a class-probability prediction reflecting its confidence score. To achieve patient-level inference, a multi-instance learning (MIL) framework (9) was adopted to aggregate slice-level outputs into comprehensive feature vectors that captured both intra- and inter-slice variability. Although the MIL stage operates at the patient level, the patch-level CNN was trained under true patch-level supervision rather than weakly assigning patient labels to all tiles. Specifically, diagnostically relevant regions were manually delineated by board-certified pathologists on the WSIs, and patch labels were generated based on these expert annotations. To ensure annotation reliability, the delineations were cross-checked by multiple readers and discrepancies were resolved by consensus. Patient-level labels were retained only for the downstream MIL aggregation step, where patch-level outputs were summarized into patient-level representations. These aggregated embeddings were subsequently refined via feature selection procedures to reduce redundancy and enhance discriminative power (**Supplementary 1**).

Similarly, after patch-level training, predicted probabilities and class labels were generated for each patch. These outputs were integrated through the MIL framework to convert patch-level predictions into WSI-level feature representations. Feature selection was again applied to these aggregated MIL features to further improve robustness and minimize redundancy.

#### Pathomics signature

Pathomics Signature was constructed from the optimized MIL-derived feature set using multiple machine-learning classifiers, including Support Vector Machine (SVM), Random Forest, and ExtraTrees (18). Model hyperparameters were tuned via grid search combined with five-fold cross-validation within the training cohort. The resulting Pathomics Signature encapsulates rich histopathological information derived from WSIs and provides a robust, generalizable representation for downstream CIP prognostic modeling.

### Clinical utilization

#### Clinical signature

All available clinical variables were first used to construct a standalone clinical prediction model. In parallel, univariable and multivariable analyses were performed to identify clinically meaningful predictors. Variables with significant associations in univariable analysis (*p* < 0.05) were carried forward, and those that remained significant in the multivariable model were ultimately retained to define the final Clinical Signature. These selected factors were then incorporated, together with Pathomics Signature, to build a Combined Model aimed at leveraging complementary clinical, imaging, and pathological information for improved CIP prediction.

We used two independent test groups to test our model, and also used receiver operating characteristic curve analysis to see how it performed. Finally, the Area Under the Curve (AUC) has become our main criterion to measure the effect of the model.

### Statistical analysis

To determine if our continuous clinical variables were normally distributed, we applied the Shapiro-Wilk test as our primary statistical check. Variables that clearly satisfied the normality assumption were analyzed using Student’s t-test, while those that failed to meet this criterion were instead analyzed using the Mann-Whitney U test. Chi-square (χ²) tests were employed to evaluate categorical variables. Baseline patient characteristics are summarized in **Table 1**. Our statistical analyses and machine learning workflows were executed using Python (version 3.7.12), OnekeyAI (version 3.3.5), and scikit-learn (version 1.0.2). The heavy lifting of model training and inference was handled by an NVIDIA RTX 4090 GPU, utilizing MONAI (version 0.8.1) and PyTorch (version 1.8.1) frameworks.

**Table 1:** Baseline of clinical features.

Feature name	Train	Val	Test1	Test2	*p*-value
Age	69.28 ± 7.75	67.64 ± 7.78	66.67 ± 6.72	68.45 ± 7.87	0.331
BMI	21.80 ± 3.35	20.97 ± 2.59	21.86 ± 2.21	20.89 ± 2.67	0.286
Gender					0.375
*Female*	20(15.50)	14(25.00)	5(9.80)	6(10.00)	
*Male*	109(84.50)	42(75.00)	46(90.20)	54(90.00)	
Smoking_habits					0.668
*Never*	51(39.53)	19(33.93)	21(41.18)	21(35.00)	
*Current/former*	78(60.47)	37(66.07)	30(58.82)	39(65.00)	
ECOG					0.024
*0*	6(4.65)	3(5.36)	2(3.92)	0	
*1*	44(34.11)	5(8.92)	2(3.92)	1(1.67)	
*2*	41(31.78)	26(46.43)	18(35.29)	25(41.67)	
*3*	38(29.46)	22(39.29)	29(57.59)	34(56.67)	
History_of_lung_disease					0.17
*Never*	84(65.12)	28(50.00)	24(47.06)	40(66.67)	
*Former*	45(34.88)	28(50.00)	27(52.94)	20(33.33)	
Cancer_stage					0.815
*TNM III B*	55(42.64)	22(39.28)	29(56.87)	33(55.00)	
*TNM IV*	74(57.36)	34(60.72)	22(43.13)	27(45.00)	
Pathological_type					1
*squamocellular carcinoma*	70(54.26)	31(55.35)	34(66.67)	33(55.00)	
*adenocarcinoma*	59(45.74)	25(44.65)	17(33.33)	27(45.00)	
Therapeutic_regimen					1
*Single ICI*	52(40.31)	23(41.08)	22(43.14)	45(75.00)	
*ICIs combination therapy*	77(59.69)	33(58.92)	29(56.86)	15(25.00)	
Driver_oncogene_mutations					1
No	129(100.00)	56(100.00)	51(100.00)	60(100.00)	
PD_L1_expression					0.01
*TPS<1%*	57(44.19)	8(14.28)	6(11.76)	14(23.33)	
*TPS 1%-49%*	28(21.71)	17(30.36)	5(9.80)	14(23.33)	
*TPS≥50%*	11(8.52)	5(8.93)	5(9.80)	1(1.67)	
*Not detected*	33(25.58)	26(46.43)	35(68.64)	31(51.67)	
RBC	4.08 ± 0.56	4.18 ± 0.53	4.32 ± 0.55	3.99 ± 0.49	0.379
HGB	112.36 ± 7.95	114.17 ± 7.39	113.00 ± 7.95	112.18 ± 7.19	0.29
PLT	204.83 ± 56.37	184.92 ± 55.47	214.19 ± 63.64	203.48 ± 45.63	0.079
WBC	7.28 ± 2.75	6.86 ± 1.97	7.26 ± 2.47	7.04 ± 2.05	0.417
NEUT	5.37 ± 3.44	5.03 ± 2.00	4.73 ± 2.24	4.95 ± 2.16	0.899
LYMPH	1.80 ± 2.13	1.47 ± 0.62	1.62 ± 0.52	1.58 ± 0.56	0.686
MONO	0.52 ± 0.25	0.51 ± 0.18	0.56 ± 0.20	0.65 ± 0.25	0.647
EOS	0.18 ± 0.17	0.14 ± 0.11	0.21 ± 0.23	0.33 ± 0.21	0.224
BASO	0.04 ± 0.02	0.04 ± 0.02	0.04 ± 0.01	0.04 ± 0.01	0.671
TP	69.89 ± 5.05	70.44 ± 5.74	69.59 ± 4.74	69.66 ± 3.85	0.599
ALB	38.84 ± 3.99	38.73 ± 4.18	39.03 ± 3.76	39.34 ± 3.26	0.895
TB	11.46 ± 4.15	11.50 ± 4.06	14.76 ± 5.17	11.27 ± 4.01	0.96
DB	5.93 ± 2.38	6.40 ± 2.64	8.01 ± 2.34	5.78 ± 2.21	0.303
IB	5.52 ± 3.16	5.09 ± 2.72	6.75 ± 4.40	5.50 ± 2.84	0.703
ALT	23.89 ± 15.30	23.97 ± 16.23	28.57 ± 21.26	26.22 ± 16.63	0.887
AST	23.12 ± 7.94	24.06 ± 10.05	23.67 ± 9.41	24.92 ± 9.45	0.939
LDH	219.32 ± 49.34	220.53 ± 53.05	208.29 ± 52.25	217.55 ± 55.05	0.899
HSCRP	20.35 ± 13.65	18.98 ± 15.64	16.81 ± 10.44	23.32 ± 23.33	0.346
NLR	4.50 ± 5.76	4.08 ± 2.38	3.39 ± 2.67	3.56 ± 1.94	0.686
PLR	163.85 ± 102.51	148.09 ± 79.87	143.53 ± 64.83	147.80 ± 69.54	0.495
ALI	92.29 ± 51.22	91.10 ± 41.14	111.30 ± 44.17	119.53 ± 61.20	0.775
SII	937.94 ± 1213.54	719.73 ± 426.18	730.30 ± 710.35	725.11 ± 417.94	0.593
PNI	47.86 ± 12.36	46.06 ± 6.02	47.14 ± 4.86	47.25 ± 4.72	0.972
SIINI	135.16 ± 203.84	105.94 ± 69.79	103.42 ± 120.05	101.05 ± 63.44	0.779

## Results

**Figure 1** summarizes not only the histopathology-based pipeline but also the downstream multimodal integration and clinical evaluation strategy used in this study. Specifically, the pathology model derived from WSI features was combined with the clinical signature to form a multimodal predictor for estimating the risk of CIP in unresectable advanced NSCLC. As shown in **Figure 1**, model performance and clinical utility were compared across the pathology-only, clinical-only, and multimodal models using AUC analyses and decision curve analysis (DCA), thereby linking algorithmic discrimination to potential net benefit in clinical decision-making.

After rigorous screening, a total of 296 cases were ultimately included for model development. Dataset A (n = 185) was randomly split into a training cohort (70%, n=129) and an internal validation cohort (30%, n=56). Following hyperparameter optimization, the model was refit using the full training cohort and subsequently evaluated on two independent external test sets (test set 1: n = 51; test set 2: n = 60) to assess generalizability to previously unseen data (**Supplementary Figure 1**).

### Analysis of clinical features

### Univariable analysis

For our investigation, we carried out a comprehensive univariate analysis of all clinical features, focusing on calculating the Odds Ratio (OR) and the associated p-values for each feature (19). Features that exhibited p-values less than 0.05 in this initial stage were selected for further analysis. In our real-world cohort of locally advanced or metastatic NSCLC, univariable signals (age, History of lung disease, high PD-L1 Tumor cell Proportion Score (TPS), and higher direct bilirubin with OR>1; basophils with OR<1) converge on a coherent biological theme: baseline pulmonary susceptibility plus a more immune-activated host state plus an inflamed TIME jointly increase the likelihood of CIP (**Supplementary Figure 2**). These significant variables were then utilized in the construction of the multivariable clinical model (**Table 2**).

**Table 2:** Univariable analysis of clinical features.

Feature_name	OR_UNI	OR lower 95%CI_UNI	OR upper 95%CI_UNI	p_value_UNI	OR_MULTI	OR lower 95%CI_MULTI	OR upper 95%CI_MULTI	p_value_MULTI
Age	1.237	1.154	1.327	0.001	1.069	1.001	1.142	0.097
Gender	0.889	0.754	1.046	0.233				
BMI	0.997	0.977	1.018	0.813				
History_of_lung_disease	1.187	1.043	1.35	0.03	1.131	1.024	1.249	0.042
ECOG	1.009	1.001	1.017	0.072				
Smoking_habits	1.029	0.901	1.175	0.724				
Pathological_type	1.08	0.949	1.228	0.325				
Driver_oncogene_mutations	1	1	1					
PD_L1_expression	1.088	1.036	1.143	0.006	1.021	1.013	1.029	0.009
Therapeutic_regimen	1.099	0.965	1.251	0.232				
Cancer_stage	1.107	0.972	1.26	0.196				
LYMPH	0.976	0.942	1.012	0.266				
IB	0.978	0.957	0.998	0.076				
WBC	0.984	0.96	1.01	0.304				
MONO	0.985	0.745	1.302	0.926				
NEUT	0.985	0.965	1.006	0.23				
ALB	0.992	0.976	1.008	0.392				
PNI	0.996	0.99	1.001	0.212				
NLR	0.997	0.984	1.01	0.676				
HSCRP	0.998	0.993	1.002	0.398				
PLT	0.999	0.998	1	0.253				
LDH	0.999	0.998	1.001	0.353				
AST	1	0.993	1.008	0.953				
ALT	1.001	0.996	1.005	0.816				
TP	1.005	0.992	1.017	0.537				
HGB	1.009	1.001	1.017	0.081				
TB	1.025	0.896	1.154	0.058				
DB	1.109	1.087	1.131	0.001	1.119	1.085	1.153	0.001
RBC	1.171	0.985	1.357	0.065				
BASO	0.621	0.410	0.832	0.023	0.881	0.831	0.931	0.098
EOS	0.794	0.529	1.194	0.351				
ALI	1.001	0.999	1.002	0.501				
SII	1	1	1	0.477				
SIINI	1	1	1	0.715				
PLR	1	0.999	1.001	0.915				

Attributes with p-values less than 0.05 were identified as statistically significant and as a result, these attributes were chosen for subsequent multivariate analysis: age, History of lung disease, PD-L1 TPS, direct bilirubin and basophils.

### MIL pathomics signature

#### Slice level efficiency

ResNet18 and ResNet101 showed similarly strong internal performance (validation AUC 0.927 vs 0.933), indicating that both backbones can learn discriminative patch-level representations under our training distribution (**Table 3**). However, both models exhibited marked degradation on external cohorts (test AUC 0.60–0.72), consistent with substantial domain shift across centers (scanning variation, case-mix differences, and label noise inherent to patient-level supervision for tiles). Notably, the two architectures differed in their operating characteristics: on the more challenging external set (test1), ResNet18 maintained higher sensitivity (0.694) and NPV (0.870) at the expense of specificity (0.490), whereas ResNet101 was more conservative with higher specificity (0.651) but substantially lower sensitivity (0.488). On test2, ResNet101 achieved a slightly higher AUC (0.724 vs 0.714) and specificity (0.555 vs 0.504), while ResNet18 retained higher sensitivity (0.895 vs 0.851) and Negative Predictive Value (NPV) (0.913 vs 0.891). The observed drop of patch-level AUC on external cohorts (0.60–0.72) is consistent with cross-center domain shift (stain variation and case-mix differences) and reflects that single-patch predictions are highly sensitive to local appearance changes. Importantly, our final inference is performed at the patient level via MIL: patch-level probabilities are aggregated into patient-level representations that capture the distribution of informative instances rather than relying on any single tile. This aggregation can attenuate center-specific noise by (i) emphasizing consistently predictive high-evidence regions across a WSI and (ii) averaging out spurious low-evidence tiles, leading to more stable external AUCs at the patient level. In addition, the subsequent feature selection step further reduces redundant/unstable dimensions and improves robustness across cohorts. For CIP risk stratification, where missed high-risk cases may be clinically consequential, the sensitivity profile of ResNet18 may be preferable, whereas ResNet101 may reduce false positives. According to the validation results, which are used as the basis for model selection, ResNet101 is the most slice level model for this task.

**Table 3:** Presents the patch-level accuracy and AUC scores for each model, focusing on label.

ModelName	Acc	AUC	95% CI	Sensitivity	Specificity	PPV	NPV	Cohort
Resnet18	0.955	0.989	0.9882-0.9900	0.940	0.959	0.876	0.981	train
Resnet18	0.829	0.927	0.9238-0.9303	0.787	0.923	0.957	0.664	val
Resnet18	0.529	0.626	0.6177-0.6344	0.694	0.490	0.246	0.870	test1
Resnet18	0.627	0.714	0.7092-0.7193	0.895	0.504	0.452	0.913	test2
Resnet101	0.937	0.978	0.9770-0.9797	0.893	0.951	0.848	0.967	train
Resnet101	0.830	0.933	0.9296-0.9355	0.784	0.930	0.961	0.663	val
Resnet101	0.619	0.599	0.5907-0.6082	0.488	0.651	0.251	0.841	test1
Resnet101	0.647	0.724	0.7189-0.7288	0.851	0.555	0.466	0.891	test2

#### Grad-CAM visualization

**Figure 3:**
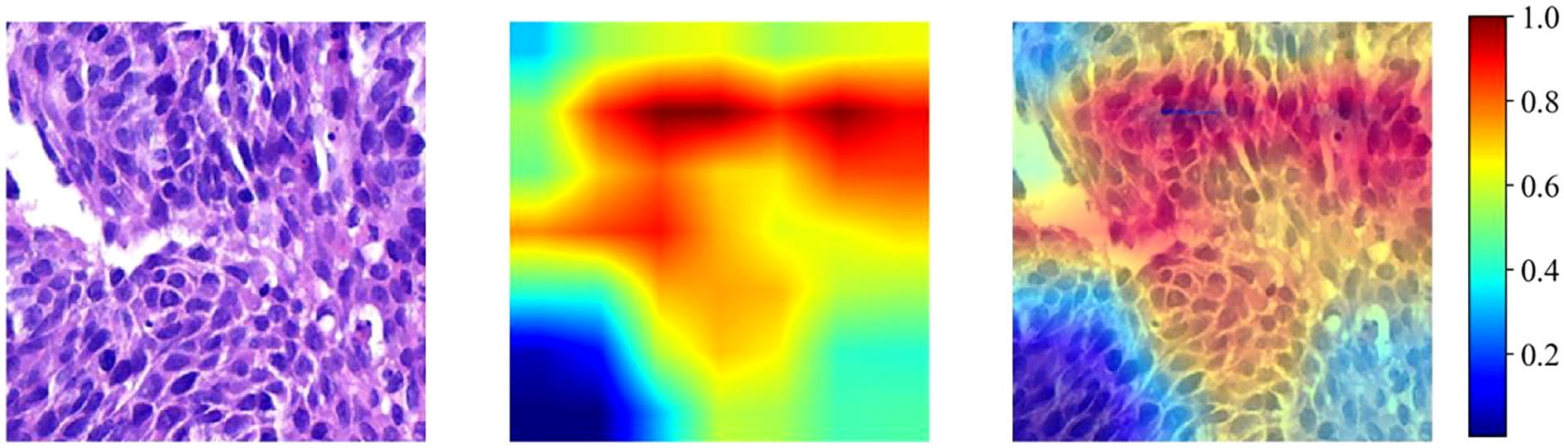
pinpoints the crucial areas in the final convolutional layer that play a pivotal role in CIP risk classification, essentially shedding light on which image components carry the most weight and infer TIME-relevant risk states.

Gradient-weighted Class Activation Mapping (Grad-CAM) (20)leverages the existing neural network architecture to produce activation maps, eliminating the need for any structural tweaks or additional training. As shown in **Figure 3**, Grad-CAM highlights the most influential regions within the final convolutional layer for CIP risk prediction, revealing which areas of the input image contribute most to the model’s decision-making process. The regions that the model attended to most strongly (highly activated areas) generally corresponded to histopathological patterns suggestive of active pneumonitis, such as infiltration by activated immune cells and potential interstitial alterations, whereas areas with dense tumor cell populations showed comparatively lower activation. This observation helps link the model’s predictions to morphologic patterns that are interpretable to pathologists and infer TIME-relevant risk states.

#### Patient level prediction

**Figure 4:**
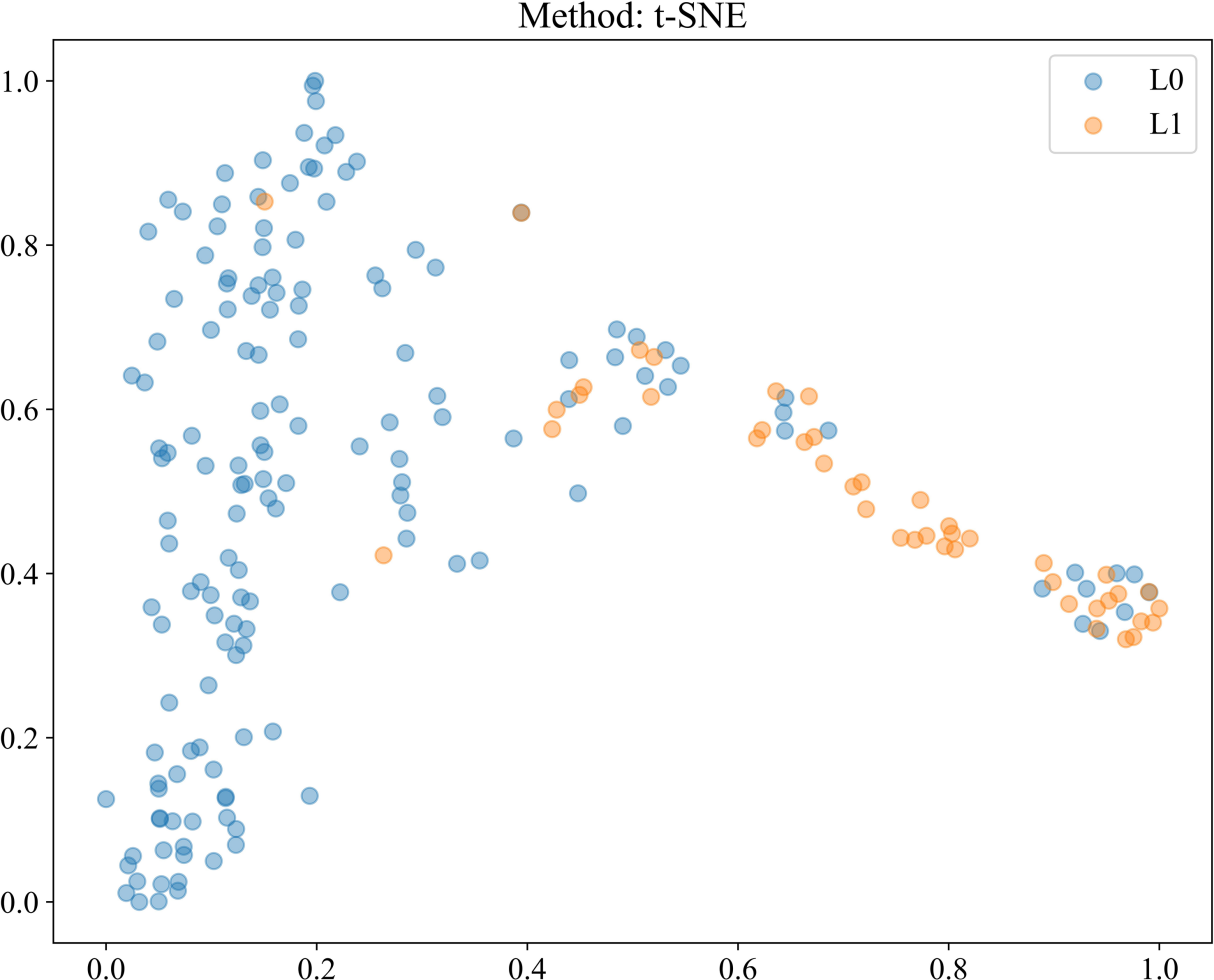
t-SNE visualization of patient-level features generated by multi-instance learning after Pearson.

The refined features were further applied in multiple machine learning models for in-depth analysis. To intuitively visualize their distribution and separability, we employed the t-Distributed Stochastic Neighbor Embedding (t-SNE) algorithm for dimensionality reduction, as illustrated in **Figure 4**, which effectively highlights the clustering patterns of different pathological classes.

**Figure 5:**
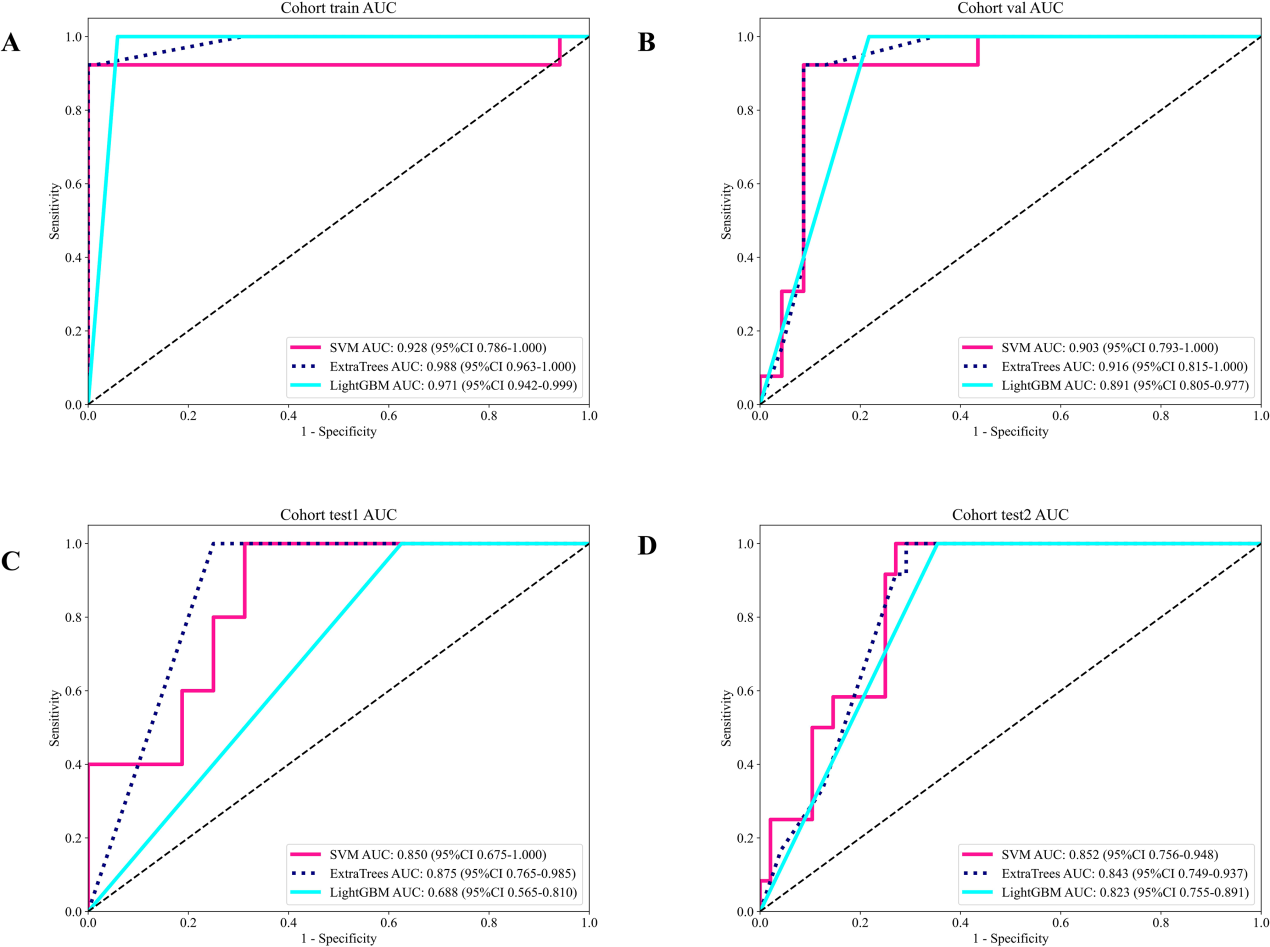
ROC curves of patient-level classifiers in each cohort: **(A)** training set; **(B)** validation set; **(C)** test 1 set;**(D)** test 2 set.

Across classifiers trained on the MIL-derived pathomics features, SVM and ExtraTrees achieved similarly strong internal performance (train/validation accuracy 0.988/0.917), whereas LightGBM showed a larger gap between internal and external results, suggesting higher sensitivity to distribution shift. In external testing, the separation between methods became evident: ExtraTrees yielded the best overall generalization on the more challenging cohort (test1 accuracy/AUC 0.810/0.875) and maintained competitive performance on test2 (0.767/0.843), while SVM was slightly lower on test1 (0.762/0.850) but marginally higher on test2 (0.783/0.852). Notably, all three approaches achieved sensitivity of 1.000 on both external cohorts at the chosen operating point, indicating that practical differences were driven primarily by specificity and PPV. Here, ExtraTrees consistently provided the most favorable trade-off (test1 specificity/PPV 0.750/0.556), reducing false positives while preserving maximal case capture, an important consideration for pretreatment CIP risk stratification (**Table 4**, **Figure 5**).

**Table 4:** Prediction performance of different signatures.

Model_name	Accuracy	AUC	95% CI	Sensitivity	Specificity	PPV	NPV	Cohort
SVM	0.988	0.928	0.786 - 1.000	0.923	1.000	1.000	0.986	train
SVM	0.917	0.903	0.793 - 1.000	0.923	0.913	0.857	0.955	val
SVM	0.762	0.850	0.675 - 1.000	1.000	0.687	0.500	1.000	test1
SVM	0.783	0.852	0.756 - 0.948	1.000	0.729	0.480	1.000	test2
ExtraTrees	0.988	0.988	0.963 - 1.000	0.923	1.000	1.000	0.986	train
ExtraTrees	0.917	0.916	0.815 - 1.000	0.923	0.913	0.857	0.955	val
ExtraTrees	0.810	0.875	0.765 - 0.985	1.000	0.750	0.556	1.000	test1
ExtraTrees	0.767	0.843	0.749 - 0.937	1.000	0.708	0.462	1.000	test2
LightGBM	0.951	0.971	0.942 - 0.999	1.000	0.941	0.765	1.000	train
LightGBM	0.861	0.891	0.805 - 0.977	1.000	0.783	0.722	1.000	val
LightGBM	0.524	0.688	0.565 - 0.810	1.000	0.375	0.333	1.000	test1
LightGBM	0.717	0.823	0.755 - 0.891	1.000	0.646	0.414	1.000	test2

### Signature comparation

**Figure 6:**
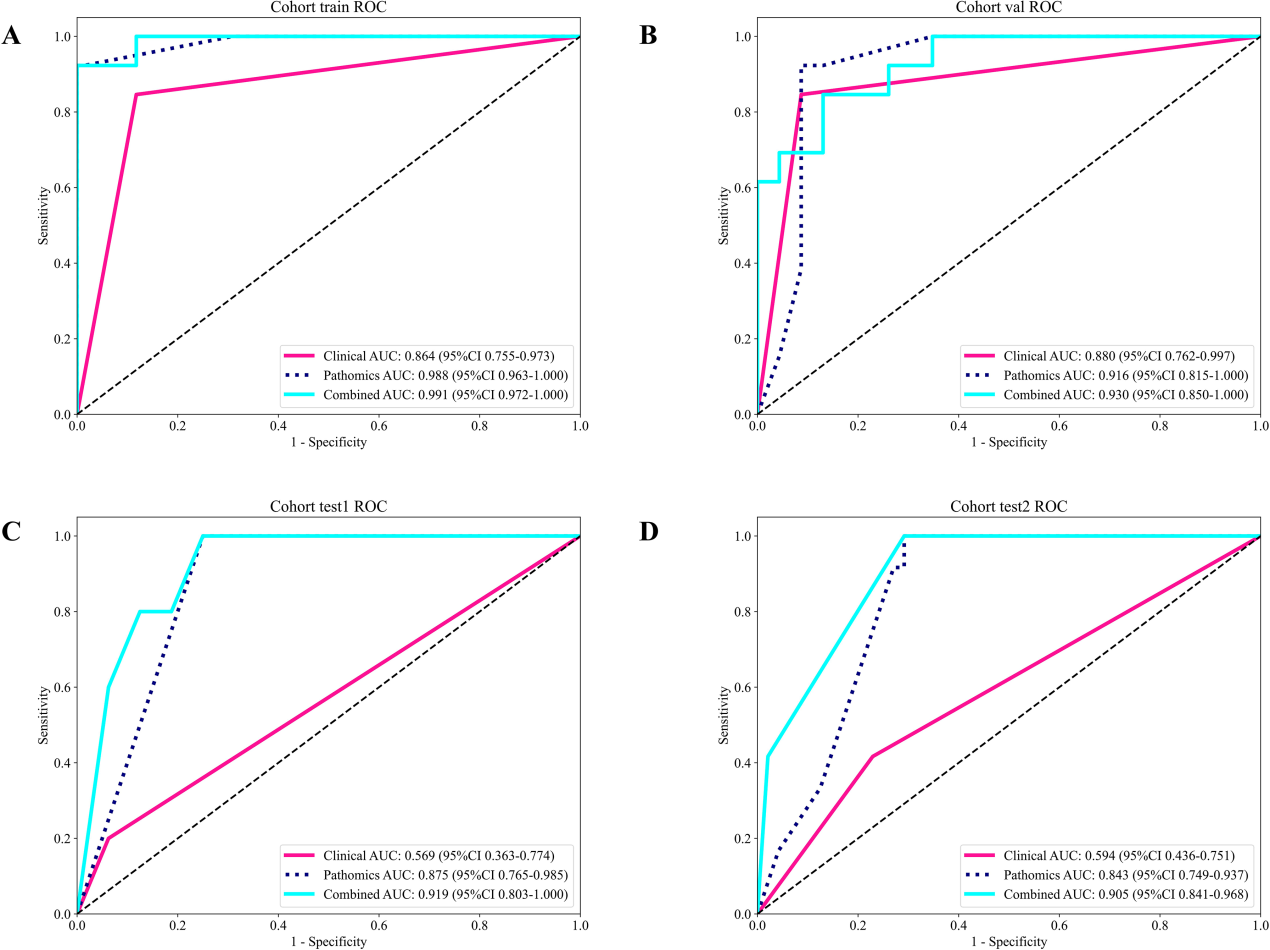
presents the data from each model measurement from each cohort and the respective ROC curves from each model executed:**(A)** training set; **(B)** validation set; **(C)** test 1 set; **(D)** test 2 set.

Within all four cohorts, the three signatures showed varying levels of predictability (**Table 5**). The validation cohort serves as the primary benchmark for models, and here the Pathomics signature had the strongest metrics with accuracy of 0.917, AUC 0.916, sensitivity 0.923, and 0.913 specificity. The Clinical signature AUC was 0.880, indicating moderate performance. The Combined model which all the information from clinical and pathology, was the best performer in the validation cohort as well with AUC 0.930 and accuracy of 0.861(**Figure 6**). This performance also reflected in the test cohort, with Pathomics also outperforming all others with AUC 0.875 (test 1) and 0.843 (test 2), while in the combined model the performance improved to AUC 0.919 (test 1) and 0.905 (test 2).

**Table 5:** Metrics on different signature.

Signature	Accuracy	AUC	95% CI	Sensitivity	Specificity	PPV	NPV	Cohort
Clinical	0.877	0.864	0.7551 - 0.9734	0.846	0.882	0.579	0.968	train
Pathomics	0.988	0.988	0.9627 - 1.0000	0.923	1.000	1.000	0.986	train
Combined	0.988	0.991	0.9722 - 1.0000	0.923	1.000	1.000	0.986	train
Clinical	0.889	0.880	0.7618 - 0.9974	0.846	0.913	0.846	0.913	val
Pathomics	0.917	0.916	0.8153 - 1.0000	0.923	0.913	0.857	0.955	val
Combined	0.861	0.930	0.8505 - 1.0000	0.846	0.870	0.786	0.909	val
Clinical	0.762	0.569	0.3634 - 0.7741	0.200	0.937	0.500	0.789	test1
Pathomics	0.810	0.875	0.7654 - 0.9846	1.000	0.750	0.556	1.000	test1
Combined	0.810	0.919	0.8026 - 1.0000	1.000	0.750	0.556	1.000	test1
Clinical	0.700	0.594	0.4362 - 0.7513	0.417	0.771	0.312	0.841	test2
Pathomics	0.767	0.843	0.7486 - 0.9371	1.000	0.708	0.462	1.000	test2
Combined	0.767	0.905	0.8412 - 0.9678	1.000	0.708	0.462	1.000	test2

The results suggest that the Pathomics signature is the single strongest predictive signature. Additionally, the integration of multi-modal signatures of Clinic and Pathomics into the Combined model shows multi-omic fusion further boosts predictive capability beyond each of the individual signatures. The clinical results are detailed in **Supplementary Figure 3**.

#### Calibration curve analysis

**Figure 7:**
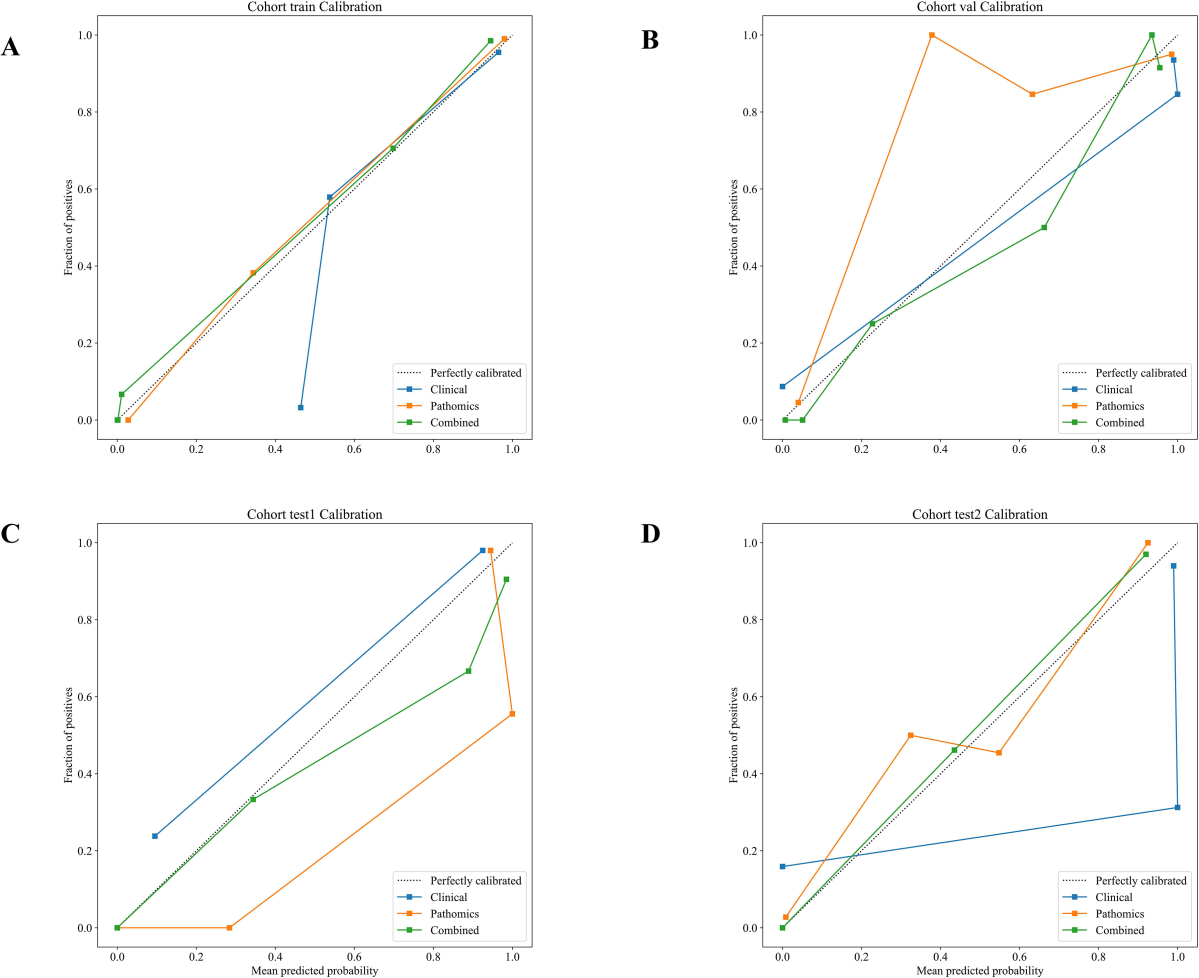
Calibration curves of different signatures in the different cohort:**(A)** training set; **(B)** validation set; **(C)** test 1 set; **(D)** test 2 set.

We assessed calibration of the combined model using calibration curves (**Figure 7**) together with the Hosmer–Lemeshow (HL) goodness-of-fit test. In the HL test, a non-significant *p*-value indicates no evidence of adequate calibration. The HL *p-*values for the combined model in the train, validation, test1, and test2 cohorts were 0.338, 0.609, 0.756, and 0.588, respectively, suggesting no detectable miscalibration in these cohorts. Given that HL results can be sensitive to sample size and grouping strategy, we primarily interpreted the calibration based on the shape of the calibration curves, using HL as a supportive check.

#### DeLong test

**Figure 8:**
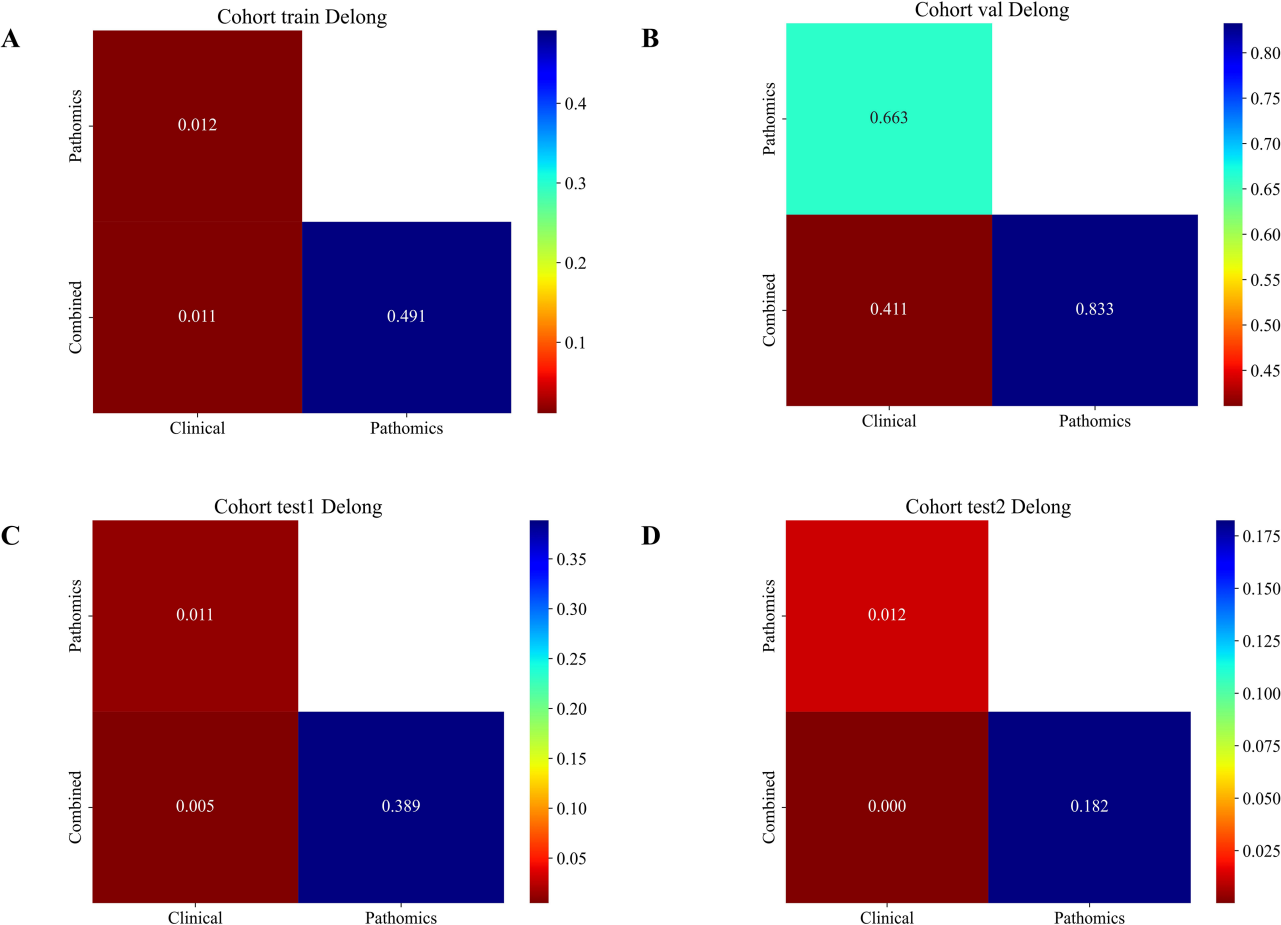
DeLong Test of different signatures:**(A)** training set; **(B)** validation set; **(C)** test 1 set; **(D)** test 2 set.

The chart below illustrates the comparative performance gains achieved by various approaches across multiple datasets, demonstrating that the integrated method consistently outshines the majority of standalone alternatives in terms of measurable improvements (**Figure 8**).

### Clinical use

#### Decision curve analysis

**Figure 9:**
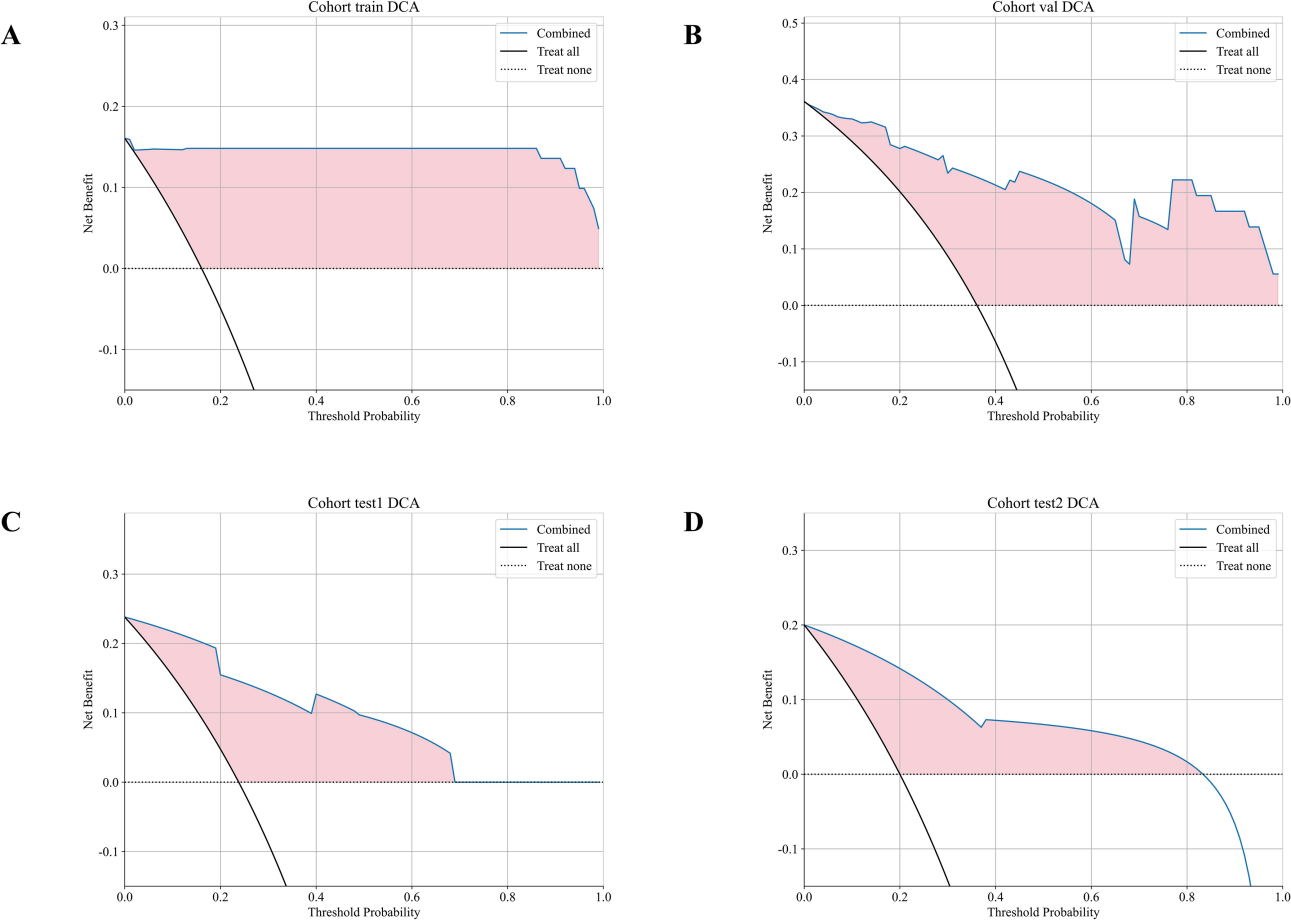
Decision curves of different signatures in the different cohort:**(A)** training set; **(B)** validation set; **(C)** test 1 set; **(D)** test 2 set.

For all cohorts, the DCA curves are displayed in **Figure 9** Through the analysis of these curves, it can be indicated that, in terms of the net benefit derived from predicted probabilities, our Combined model offers a notable advantage.

## Discussion

In recent years, with a persistent upward trend shown in both the incidence and mortality rates of lung cancer, immune checkpoint inhibitors (ICIs), remarkably, have improved the survival and quality of life of patients with unresectable, advanced NSCLC (11, 21). However, CIP remains a major toxic limitation. Once this occurs, subsequent antitumor immunotherapy can be severely affected. Moreover, over 25% of affected patients may develop respiratory failure or even death during ongoing ICI treatment (22), with a median time to CIP onset of approximately 2–3 months after ICI initiation. Most cases occur within the first six months of treatment. Nevertheless, CIP can arise within hours of the first ICI dose, and it may also develop after treatment discontinuation (3, 23, 24). CIP lacks specific serum biomarkers, and its clinical symptoms are nonspecific, with dry cough, exertional dyspnea, and reduced exercise tolerance being typical presentations. Fatigue, fever, and chest pain may also occur and CIP histopathological findings are also not pathognomonic (5, 23, 25). Therefore, CIP is usually diagnosed by exclusion and requires the integration of medical history, imaging, serology, microbiology, bronchoscopy, and multidisciplinary discussion. If CIP is identified and treated early, the prognosis is generally favorable. Some patients may even undergo subsequent immunotherapy rechallenge (1, 26). However, substantial challenges remain in constructing a more precise platform for predicting treatment response and adverse events.

In our real-world cohort of locally advanced or metastatic NSCLC, univariable associations (age, pre-existing lung disease, high PD-L1 TPS, and higher direct bilirubin with OR>1; basophils with OR<1) together suggest that CIP susceptibility may reflect the combined effects of baseline pulmonary vulnerability, host inflammatory status, and tumor immune context (27). Advanced age has been reported as a CIP risk factor in lung cancer studies and may be related to immunosenescence and chronic low-grade inflammation (28, 29), while pre-existing pulmonary disease (pulmonary fibrosis, chronic obstructive pulmonary disease) is consistently linked to CIP and may lower the threshold for immune-mediated lung injury after PD-1/PD-L1 blockade (30, 31). Higher PD-L1 expression may serve as a surrogate for an immune-infiltrated TIME and interferon-related signaling, although evidence across cohorts is not fully uniform (28, 32, 33). Direct bilirubin is not a recognized CIP-specific marker and may reflect comorbidity or systemic inflammation; therefore, it should be interpreted as exploratory. The inverse association with basophil count lacks direct clinical corroboration but may indicate baseline myeloid-lineage remodeling relevant to susceptibility. Notably, the clinical model showed a marked performance drop in the external Test 1 and Test 2 cohorts (AUC = 0.569/0.594), whereas the pathomics model exhibited comparatively better stability across the cohorts. This discrepancy is consistent with the notion that structured clinical variables are more vulnerable to cross-center distribution shifts and heterogeneous missingness mechanisms, even though we used stratified splitting (70% training and 30% internal validation) and five-fold cross-validation with grid search during model development. In particular, differences in clinical testing practices and documentation can substantially alter feature availability and the effective information content at inference time. For example, the proportion of PD-L1 status recorded as “Not detected” increased from 25.58% in the training set and 46.43% in the internal validation set to 68.64% in Test 1 (and 51.67% in Test 2), suggesting a cohort-dependent missingness pattern that may impair clinical model generalization. We noted that the proportion of “Not detected” PD-L1 results differed across cohorts, and such missingness patterns may partially reflect center-specific practice. Therefore, missingness itself could carry implicit center information, which may contribute to apparent predictability and affect cross-center generalization. Moreover, variations in treatment pathways (regimen composition and clinical workflow) across centers may further amplify this instability. Under a host–lung substrate–TIME” framework, these clinical factors describe host and lung background risk (34), whereas our H&E-based pathomics is intended to provide complementary tissue-level information on TIME to improve pretreatment risk stratification.

Therefore, we developed and validated a multicenter multimodal prediction model integrating baseline clinical biomarkers with H&E-based WSI pathomics using multi-instance learning (MIL) to estimate the probability of CIP in patients with advanced NSCLC treated with PD-1/PD-L1 inhibitors. Current biomarker discovery paradigms range from conventional histopathological assessments to interdisciplinary mining of multimodal data sets. Pathomics represents an innovative cross-disciplinary domain merging digital pathology with artificial intelligence. With advanced whole-slide scanners, standard H&E slides can be digitized into WSIs. The histological features can then be automatically extracted and categorized. These features were transformed into computable variables. Subsequently, complex algorithms can be applied to tasks such as cancer classification and prognostic predictions (35, 36). Among the most widely used techniques for pathology image analysis, convolutional neural networks (CNNs) remain the most central. CNNs consist of convolutional filters that apply local transformations across images with shared parameters. As a result, efficient parameterization was achieved, and a degree of translation invariance was obtained. Pooling layers are typically inserted to downsample intermediate feature maps. Fully connected layers then generate the final representations from the flattened features. Model optimization is performed by iteratively updating weights and biases to minimize a loss function. Compared with traditional image classification approaches, CNNs are advantageous for end-to-end learning and flexible pattern discovery from image data (8). Kao Y-S et al. (36) investigated deep learning–based computational pathology (deep pathomics) to predict treatment response in stage III NSCLC. Their study analyzed 35 digital tissue samples, including biopsy samples and surgical samples, all from patients diagnosed with stage IIIA/IIIB non-small cell lung cancer. The response to treatment is determined by weekly CT scans, which track the changes of target volume during radiotherapy and chemotherapy, thus dividing patients into responders and non-responders. In order to evaluate the performance, the researchers tested five pre-trained convolutional neural networks: AlexNet, VGG, MobileNet, GoogLeNet and ResNet. Among them, GoogLeNet has the best performance, high specificity and stable sensitivity. Generally speaking, these findings show that pathology can identify disease patterns by combining traditional pathological semantics with clinical data. It can also play a complementary role by interacting with different omics layers. These methods can detect subtle lesions that are imperceptible to the naked eye and may reduce subjectivity. Consequently, they can support screening, diagnosis, differential diagnosis, and prognosis assessment. In addition, Vanguri et al. (37)reported that a multimodal model outperformed the unimodal models for CIP prediction. Thus, their results provide indirect support for our findings. In the present study, pretreatment heterogeneity and baseline TIME features derived from lung tumor WSIs were leveraged to predict CIP following ICIs therapy. This approach may guide the management of post-ICI pneumonitis and facilitate the timely adjustment of antitumor strategies. Importantly, this modeling framework is innovative compared to prior studies. Compared with the unimodal models that rely on pretreatment serological inflammatory indices for CIP risk estimation, our pathology-clinical multimodal fusion model really steals the show, boasting superior performance with AUC scores of 0.930, 0.919 (test 1) and 0.905 (test 2) in the validation and test sets, respectively. The enlightenment of this model is that it needs to be adjusted according to different clinical scenarios (such as giving priority to high NPV for excluding high risk vs. increasing the threshold to increase PPV for intensive intervention), and combined with the decision curve (DCA) to give the explanation of net benefit interval and the principle of threshold selection. Suggestions for risk stratification management of possible CIP: Low risk: follow-up according to routine plan; Medium risk: shorten the follow-up interval, improve the frequency of symptom screening, and review HRCT earlier if necessary; High risk: early treatment of intensive respiratory and imaging combined follow-up; when mild respiratory symptoms occur, the differential diagnosis process (infection exclusion, imaging evaluation, bronchoscopy if necessary) is initiated at a lower threshold. Finally, whether prophylactic medication should strictly follow the current guidelines and clinical judgment. Meanwhile, the notion of “TIME-related risk states” or latent subtypes is increasingly used to reconcile clinical heterogeneity (38) when direct mechanistic measurement is unavailable. In large adolescent cohort studies (39–41), multimodal data integration has been leveraged to delineate transdiagnostic dimensions and subtype-like patterns, thereby improving interpretability and prediction beyond coarse diagnostic labels (42–44). In this context, we use the term “TIME-related risk states” in an analogous, strictly phenotype-based sense: the model infers risk-relevant patterns from routine H&E and clinical variables, while mechanistic and cell-type–resolved validation remains an important direction for future work.

Several limitations should be acknowledged. First, this study was retrospectively conducted using data from three hospitals in China. Substantial inter-center differences in CIP incidence were observed, and the overall sample size remained limited, which may affect the robustness and generalizability of the findings. Therefore, prospective validation in larger cohorts and additional centers is warranted. Second, baseline chest CT images were not included in the current multimodal framework. Future work may benefit from integrating clinical, imaging, and pathological modalities under a unified modeling strategy (Transformer-based architectures with attention-based fusion) (45). Modality-specific feature learning will be extended to cross-modal representation learning (46, 47) to enable more comprehensive risk assessment and to better characterize radiologic–pathologic patterns underlying CIP. Third, although direct bilirubin (DB) and basophils (BASO) emerged as informative variables in multivariable modeling, they should be interpreted as exploratory and non-specific associative markers rather than direct causal determinants of CIP. These signals may act as proxies for underlying host conditions (systemic inflammatory status, immune–metabolic background, and comorbidity burden) and may also be influenced by residual confounding inherent to real-world retrospective data (baseline hepatobiliary dysfunction, tumor burden, concomitant medications, and other unmeasured clinical factors). Accordingly, our interpretation of DB and BASO is framed as testable hypotheses, and mechanistic validation (such as immune phenotyping, cytokine profiling, immunohistochemistry, or spatial-omics analyses) lies beyond the scope of the present retrospective study and should be addressed in future prospective investigations. Ultimately, a more comprehensive predictive model will be established, and deeper insights into the radiologic-pathologic characteristics and their pathophysiological and biological foundations will be obtained. While our cohort was restricted to PD-1/PD-L1 inhibitors, detailed treatment-context variables (ICI class distribution across centers, line of therapy, monotherapy versus combination regimens, and post-chemoradiotherapy consolidation status) were not fully captured or were imbalanced, which may introduce residual confounding and limit the reliability of clinical interpretation in specific real-world scenarios and the generalizability of our findings to CTLA-4–based strategies remains uncertain.

## Conclusion

In summary, age, History of lung disease, PD-L1 TPS, and direct bilirubin (DB) and basophil count (BASO) were identified as clinical biomarkers of CIP. A multi-instance learning-based pathomics signature derived from pretreatment H&E whole-slide images, when integrated with baseline clinical variables, markedly improved CIP prediction compared with unimodal models. This multimodal framework provides a practical, scalable approach to infer TIME-relevant risk states from routine pathology and supports proactive surveillance and individualized immunotherapy strategies, with a clear path toward future integration of radiomics and spatial multi-omics for pathway-level interpretability.

## Data Availability

The datasets presented in this study can be found in online repositories. The names of the repository/repositories and accession number(s) can be found in the article/**Supplementary Material**.

## Glossary

NSCLC: Non-small cell lung cancer
CIP: Checkpoint inhibitor pneumonitis
irAEs: immune-related adverse events
AI: Artificial intelligence
TIME: tumor immune microenvironment
H&E: Hematoxylin and eosin
CNN: Convolutional neural networks
MIL: multi-instance learning
WSI: Whole-slide imaging
LR: Logistic regression
ROC: Receiver Operating Characteristic
AUC: Area under the curve
ICI: Immune checkpoint inhibitor
IL-6: Interleukin-6
PD-1: Programmed death 1
PD-L1: Programmed death ligand-1
CTLA-4: Cytotoxic T-lymphocyte-associated protein 4
HIS: hospital information system
BMI: Body Mass Index
ECOG: Eastern Cooperative Oncology Group
RECIST: Response Evaluation Criteria in Solid Tumors
TPS: Tumor cell Proportion Score
TNM: Tumor Node Metastasis
HRCT: High-resolution Computed Tomography
CNN: convolutional neural network
SGD: stochastic gradient descent
SVM: Support Vector Machine
OR: Odds Ratio
CI: Confidence Interval
RBC: Red Blood Cell Count
HGB: Hemoglobin
PLT: Platelet Count
WBC: White Blood Cell Count
NEUT: Neutrophil Count
LYMPH: Lymphocyte Count
MOMO: Monocyte Count
EOS: Eosinophil Count
BASO: Basophil Count
TP: Total Protein
ALB: Albumin
TB: Total Bilirubin
DB: Direct Bilirubin
IB: Indirect Bilirubin
ALT: Alanine Aminotransferase
AST: Aspartate Aminotransferase
LDH: Lactate Dehydrogenase
HSCRP: Hypersensitive C-reactive protein
NLR: Neutrophil-to-Lymphocyte Ratio
PLR: Platelet-to-Lymphocyte Ratio
NPV: Negative Predictive Value
PPV: Positive Predictive Value
Grad-CAM: Gradient-weighted Class Activation Mapping
t-SNE: t-Distributed Stochastic Neighbor Embedding
HL: Hosmer-Lemeshow
DCA: Decision Curve Analysis.
