# Supplementary material for: Inferring tumor immune microenvironment -related risk states from pretreatment H&E pathomics and clinical biomarkers to predict checkpoint inhibitor pneumonitis in advanced NSCLC: a multicenter multimodal study
Source: Front Immunol. 2026 Feb 19;17:1792179. doi: 10.3389/fimmu.2026.1792179 (PMC12960525; doi:10.3389/fimmu.2026.1792179)
Supplement: Supplementary file 1 [file DataSheet1.docx]

**Supplementary**

**1A. Patch Level Model Training Details**

In order to increase the model's versatility with a wide range of patient groups, which often exhibit considerable diversity, we resorted to transfer learning. This entailed kickstarting the model with weights that had been pre-trained on the ImageNet database, a move that proved instrumental in expanding the model's flexibility in handling different types of data. A key point in our strategy is to accurately adjust the learning rate, which is very important to improve the overall applicability of the model. For this purpose, we utilized the cosine decay learning rate algorithm, which is delineated as follows:


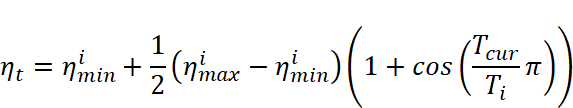


In this configuration, the notation
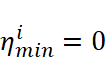
 specifies the minimum learning rate, and
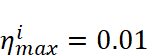
 indicates the maximum learning rate. Additionally,
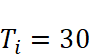
 represents the number of iteration epochs in the training process. Other key hyperparameter settings include the use of SGD (Stochastic Gradient Descent) as the optimizer, and softmax cross entropy as the loss function.

**1B. Multi-Instance Learning-Based Feature Fusion**

In our research, we decided to use the method of multi-instance learning to combine features, mainly to improve the prediction ability of the model. The principle of this technology is to combine different data or individual examples in a case to form a complete feature file. We find that this strategy is particularly important for the accurate analysis and prediction of complex medical diagnosis. In the next part, we will explain in detail the specific steps and methods we use in the process of feature integration:

1. **Patch Prediction**: We tapped into the Resnet50 model to forecast each patch, yielding respective likelihoods and labels, which we referred to as
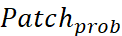
 and
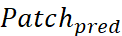
, respectively. The prediction probabilities were retained to two decimal places.
2. **Multi Instance Learning Feature Aggregation:**
   1. **Histogram Feature Aggregation**:
      1. We categorized each unique figure into a "bin" and tabulated the prevalence of each data type within these bins.
      2. The frequencies of
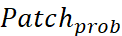
 and
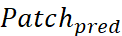
 falling into each bin were tallied.
      3. Features were subject to min-max standardization.
      4. This process resulted in the generation of
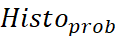
 and
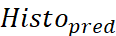
.
   2. **Bag of Words (BoW) Feature Aggregation**:
      1. Initially, a dictionary was created by identifying unique elements within
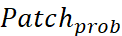
 and
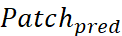
.
      2. Every patch was subsequently transformed into a vector format, documenting how often each dictionary element appeared within that specific patch.
      3. We then put these vectors through the wringer with a Term Frequency-Inverse Document Frequency (TF-IDF) transformation, which helped spotlight rarer yet more meaningful characteristics.
      4. This resulted in a BoW feature representation for each patch, encapsulating both the presence and significance of features within a patch.
      5. The final BoW features, denoted as
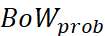
 and
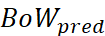
, offered a comprehensive and weighted representation of the patches, suitable for subsequent analytical processes.
3. **Feature Early Fusion**: In the last step of our multi-instance learning feature fusion process, we should combine the features obtained before:
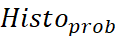
,
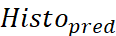
,
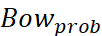
, and
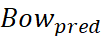
. The formula for this merging process is laid out like this:In order to make this idea come true, we use a cool function merging technique to represent it with a symbol
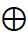
. This technique can merge several groups of different functions into a total large function vector. The formula for the merger process is written as follows:


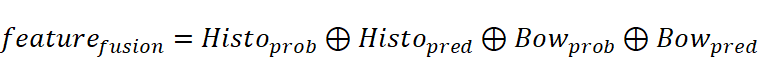


**2A Convolutional neural network architecture**

We used a ResNet-101 model pretrained on the ImageNet dataset as the feature extractor. The architecture is summarized as follows.

**Convolutional configuration:** ResNet-101 contains 101 learnable layers, including 100 convolutional layers within the residual blocks. Specifically, it consists of: (i) one initial 7×7 convolutional layer; (ii) four residual stages with 3, 4, 23, and 3 residual blocks, respectively; and (iii) three convolutional layers (1×1, 3×3, 1×1) within each residual block. In total, the network includes 100 convolutional layers followed by a global average pooling layer.

**Activation and normalization:** ReLU is applied after each convolutional layer. Batch normalization is used within each residual block prior to the shortcut (skip) connection.

**Feature extraction:** The original classification head was removed, and the convolutional backbone was retained as the feature extractor. For each input image patch (224×224 pixels), the network outputs a 1,024-dimensional feature vector corresponding to the channel dimension of the final convolutional feature map.

**Loss function and optimization**

**Loss function:** We used the cross-entropy loss, defined as

$$\mathcal{\mathcal{L}}=-\frac{1}{N}\sum_{i=1}^{N} \log\left( \frac{\exp\left( z_{i,y_{i}} \right)}{\sum_{j=1}^{C} \exp\left( z_{i,j} \right)} \right)$$

where$N$is the number of samples,$C$is the number of classes,$y_{i}$ is the ground-truth label for sample $i$,and$z_{i,j}$ denotes the model output (logit) for sample $i$ in class $j$ .

**Supplementary 2:**

This study, grounded in a retrospective analysis of clinical data, has led to the discovery that combining pre-treatment neutrophil count, lymphocyte count, platelet count, hemoglobin level, serum albumin concentration, and body mass index (BMI) into a novel composite metric—the Systemic Immune-Inflammatory Nutritional Index (SIINI), calculated as [neutrophil count × platelet count × hemoglobin level] / [lymphocyte count × BMI × serum albumin concentration].NLR, PLR, ALI, SII, PNI and were calculated by NLR = neutrophil count / lymphocyte count, PLR = platelet count / lymphocyte count, ALI = BMI × serum albumin level NLR, SII= platelet count × NLR, PNI = serum albumin level + 5 × lymphocyte count.

**Supplementary figures:**


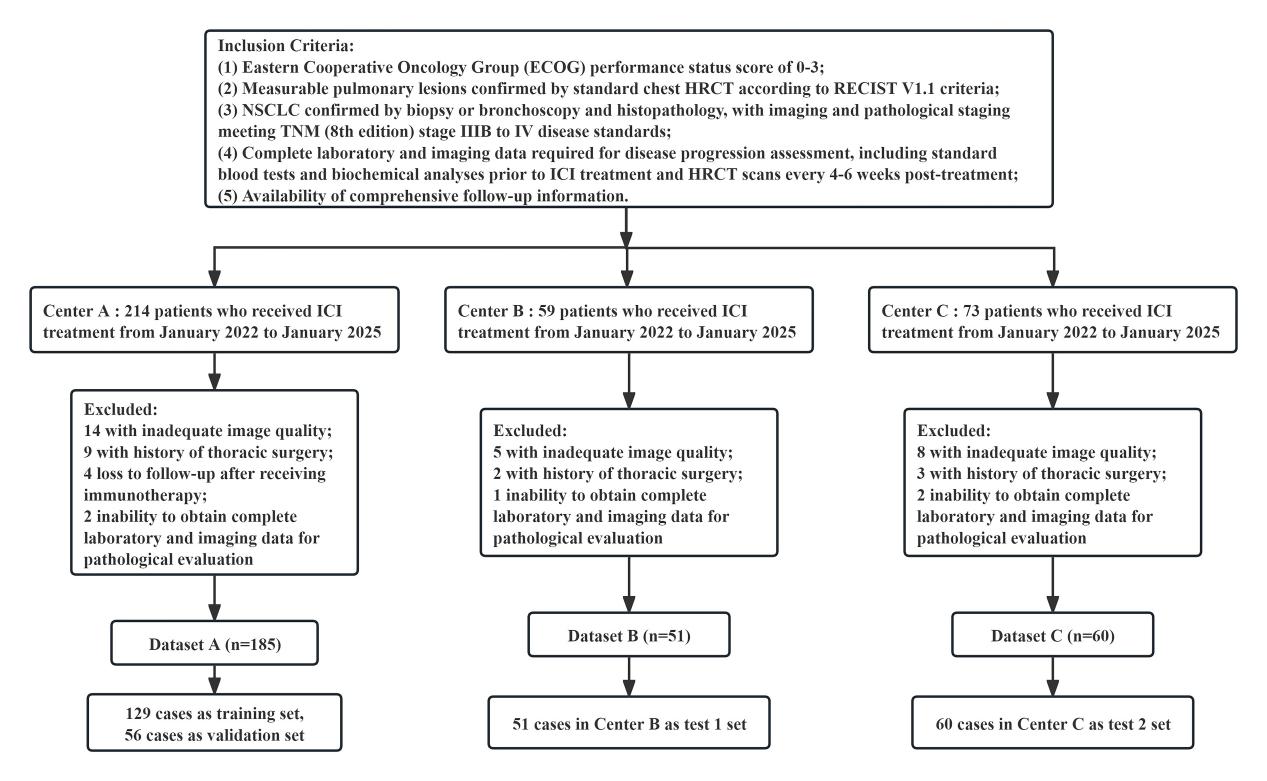


**Supplementary Figure 1. The inclusion criteria and cohort distribution of this study.**

**
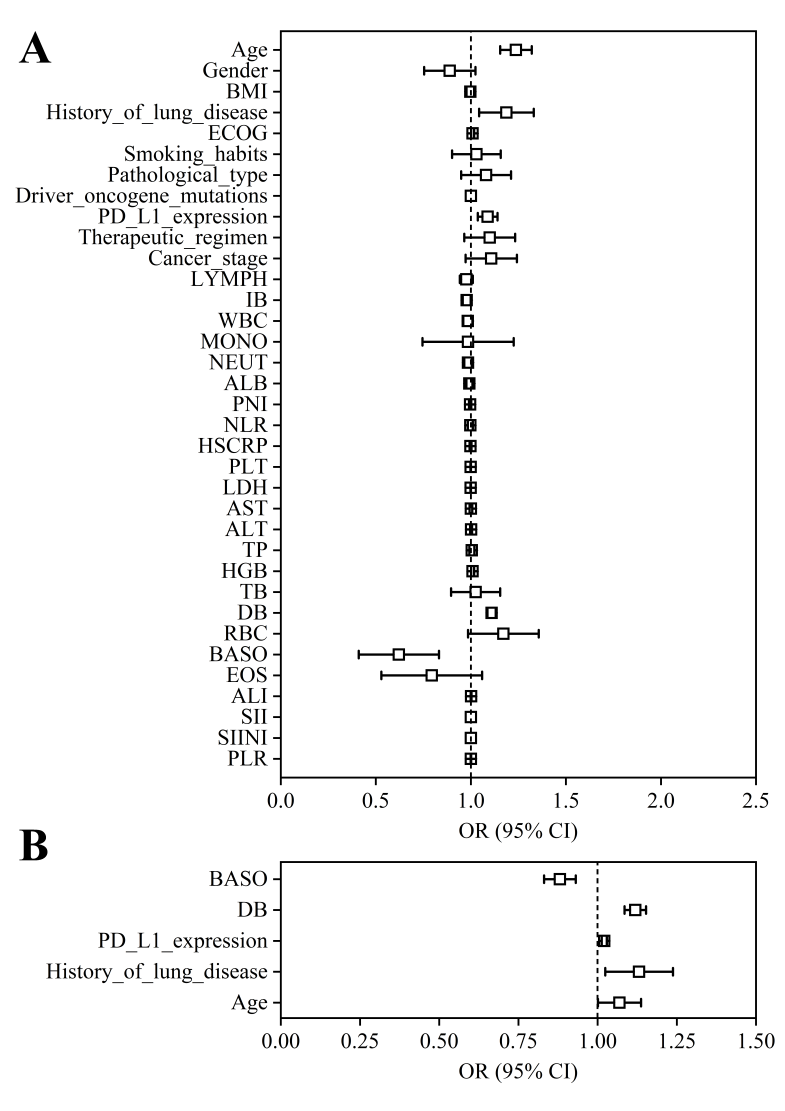
**

**Supplementary Figure 2. Univariate analysis of all clinical features.**

**
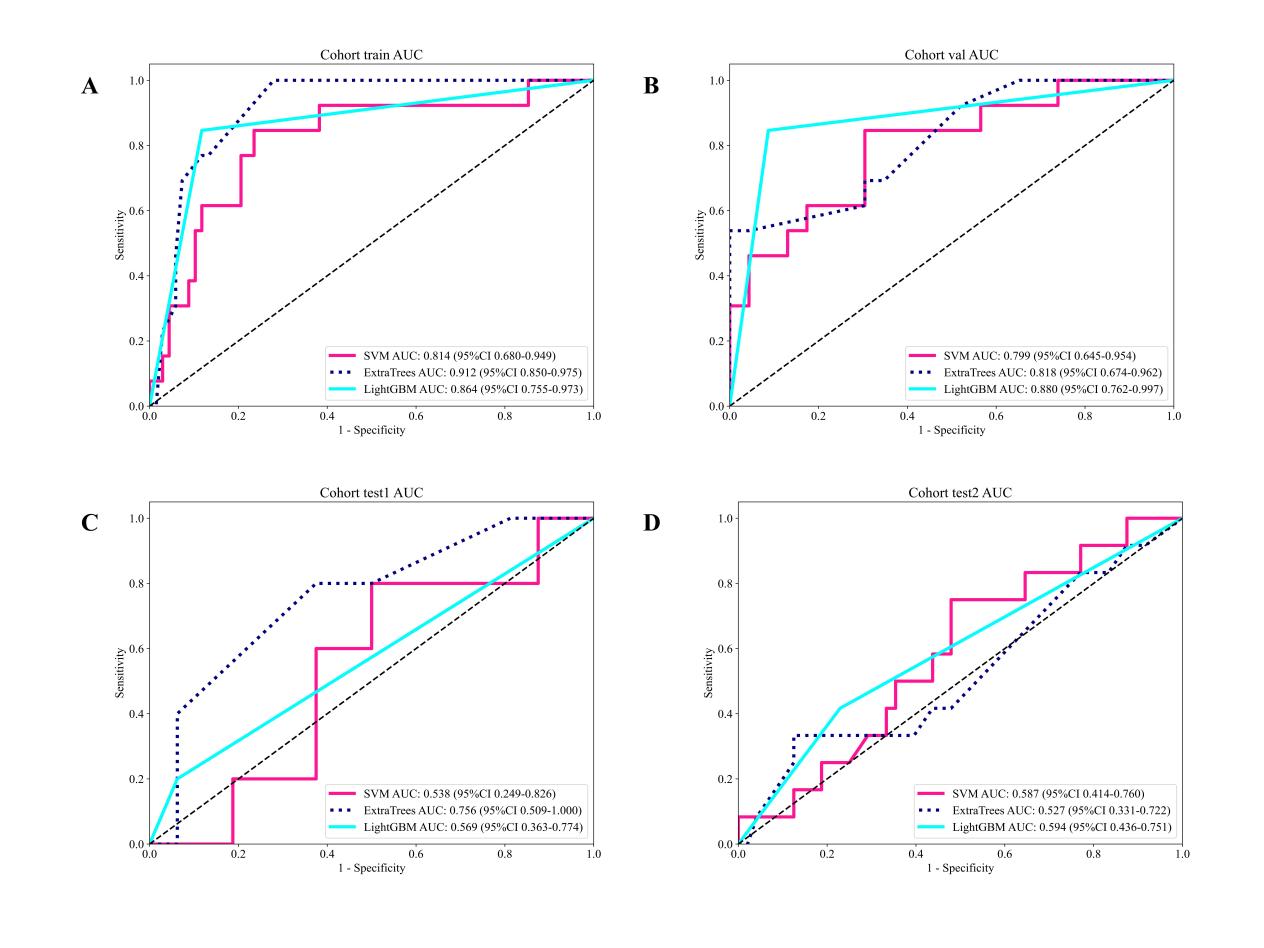
**

**Supplementary Figure 3. Metrics of the clinical model.**

**
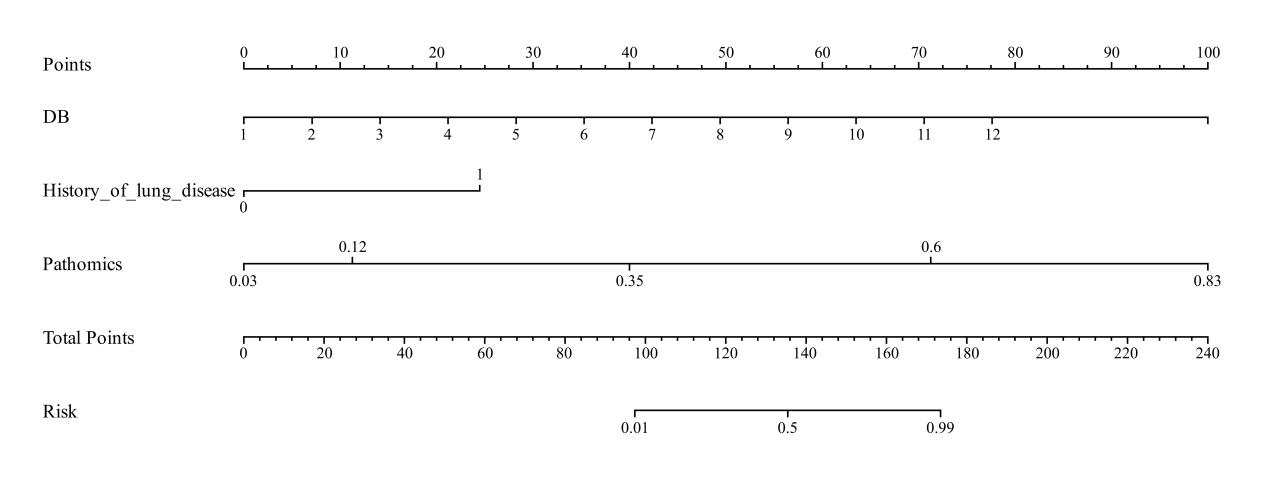
**

**Supplement Figure 4. Nomogram.**

**
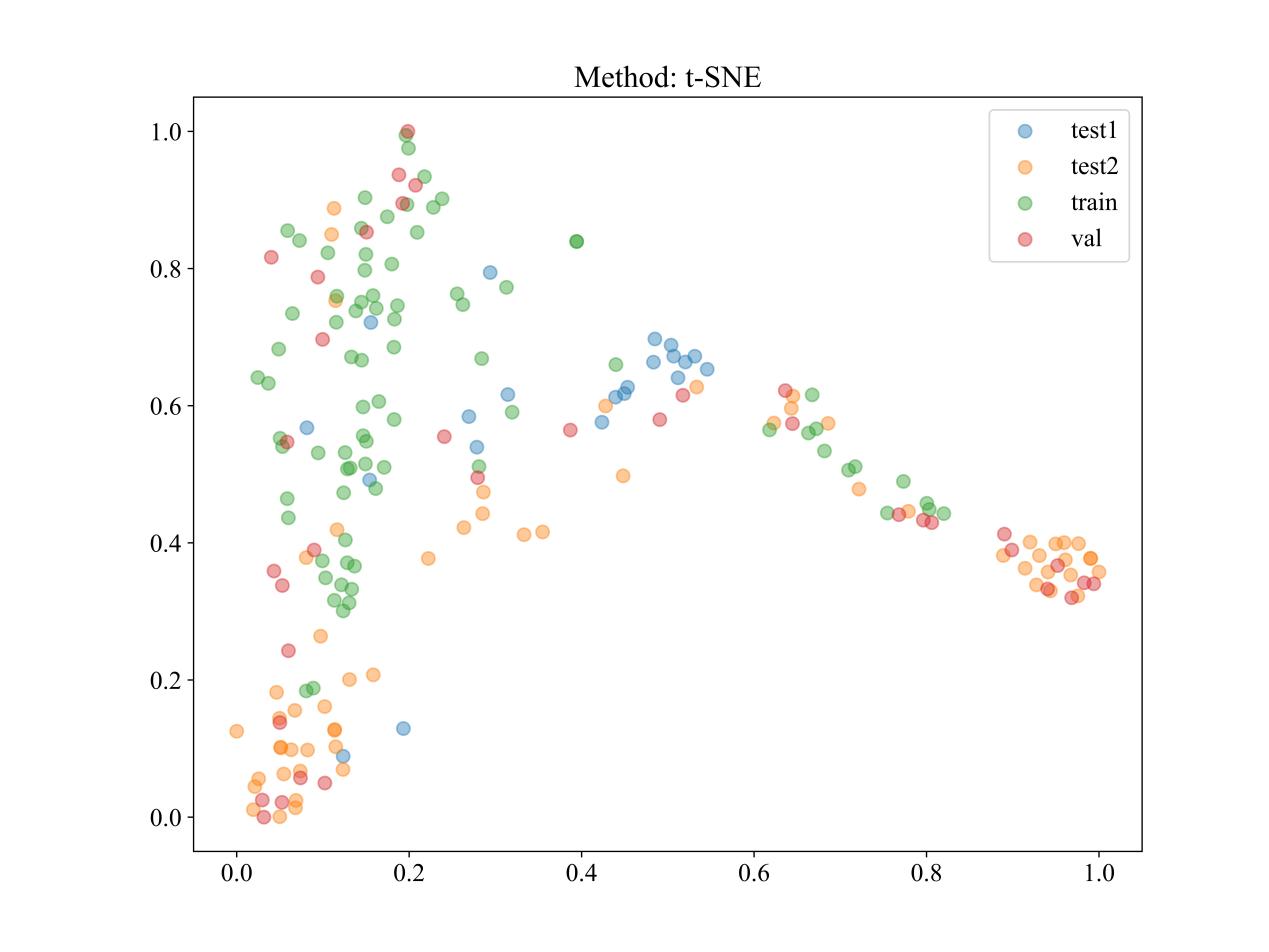
**

**Supplement Figure 5. t-SNE of different cohorts.

Notes：
The data presented in this study are available in the Supplementary Material and Zenodo DOI: 10.5281/zenodo.18310194.**

**Additional patient-level data are not publicly available due to privacy and ethical restrictions, but may be obtained from the corresponding author upon reasonable request and subject to institutional approval.**
