# Supplementary figures and images for: Inferring tumor immune microenvironment -related risk states from pretreatment H&E pathomics and clinical biomarkers to predict checkpoint inhibitor pneumonitis in advanced NSCLC: a multicenter multimodal study
Source: Front Immunol. 2026 Feb 19;17:1792179. doi: 10.3389/fimmu.2026.1792179 (PMC12960525; doi:10.3389/fimmu.2026.1792179)

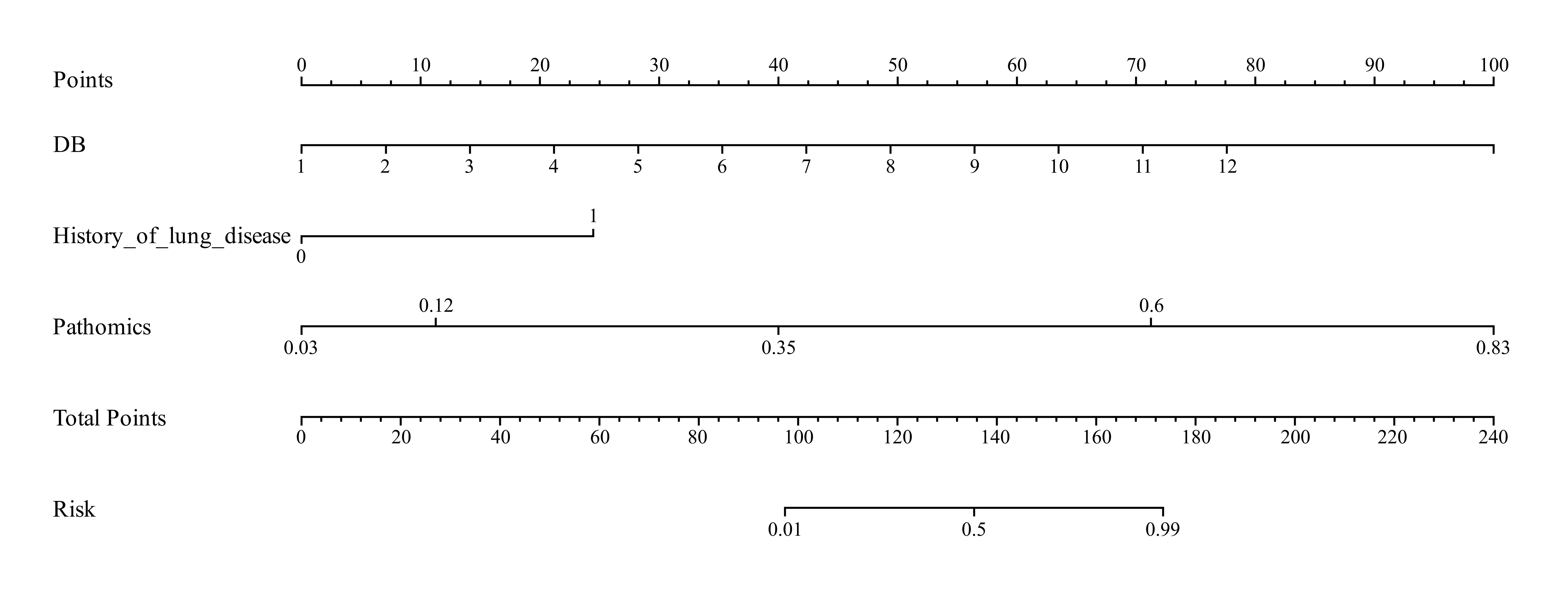

Supplement: Supplementary file 5 [file Image4.jpeg]
